# Tunable Emission Peak Position and Enhanced Thermal Stability of CsPbBr_3_ Quantum Dots via TMCS Ligand Exchange

**DOI:** 10.3390/ma19091860

**Published:** 2026-05-01

**Authors:** Chong Peng, Yutao Feng, Zhicheng Shen, Zhe Pang, Shujing Ren, Xiaoqian Wang, Yingfei Liu, Jiaqian Que, Kefeiyang Hu, Xingbo Huang, Yong Liu

**Affiliations:** State Key Laboratory of Advanced Technology for Materials Synthesis and Processing, International School of Materials Science and Engineering (ISMSE), Wuhan University of Technology, Wuhan 430070, China; 15856589663@163.com (C.P.); 18768184092@163.com (Y.F.); a2424699596@163.com (Z.S.); 15091422674@163.com (Z.P.); rshujing@163.com (S.R.); 303568@whut.edu.cn (X.W.); liu714961@163.com (Y.L.); 294599@whut.edu.cn (J.Q.); hkfy8789@whut.edu.cn (K.H.); 15251300417@163.com (X.H.)

**Keywords:** perovskite quantum dots, ligand exchange, improved thermal stability

## Abstract

All-inorganic lead halide perovskite quantum dots (QDs), featuring high photoluminescence quantum yield, narrow full width at half maximum, and solution processability, show great promise for high-color-purity displays and optoelectronic devices. Their emission peak position and stability are highly dependent on the surface coordination environment, and achieving controllable color tuning while maintaining stability without altering the primary synthetic route remains a critical challenge. Herein, we propose a facile solution-phase post-treatment strategy using TMCS, which can react with the oleate ligands on the CsPbBr_3_ QD surface while providing abundant Cl^−^ ions, thereby leading to partial halide exchange, achieving continuous tuning of the emission wavelength from 499 nm to 473 nm. The appearance of new absorption peaks in the FTIR spectra indicated the successful introduction of TMCS and the in situ generation of HCl, which led to surface etching and passivation. After being heated at 40 °C for 6 h, the TMCS-50 sample retained 39% of its initial photoluminescence intensity, while the pristine CsPbBr_3_ QD sample retained only 8%, demonstrating that TMCS treatment significantly improves the thermal stability of the CsPbBr_3_ QDs.

## 1. Introduction

All-inorganic lead halide perovskite quantum dots (QDs) have attracted extensive interest for displays [1,2,3], lighting [4,5,6], and photodetectors [7,8,9] due to their high PLQY, narrow full width at half maximum (FWHM), and solution processability [10]. Compared with conventional II-VI quantum dots, CsPbBr_3_ QDs can deliver “purer” visible emission and broader color-gamut coverage [11]. Meanwhile, influenced by nanoscale quantum-confinement effects and surface chemistry, their emission performance and peak position are highly sensitive to particle size, composition, and the surface ligand environment [12,13,14,15]. Developing a simple and controllable approach to tune the emission wavelength without changing the primary synthetic route, while simultaneously improving stability, remains a key challenge for practical applications of perovskite quantum dots.

At present, emission-wavelength tuning of CsPbBr_3_ QDs is mainly achieved through halide-composition engineering (Br/Cl or Br/I exchange) [16], size regulation by controlling reaction temperature and time, or post-treatment-induced surface reconstruction via ligand introduction [17,18]. Among these strategies, post-treatment has received growing attention because it is mild, causes minimal perturbation to the original system, and is amenable to scale-up and process integration [19]. Ligand exchange-based surface passivation can directly modulate the surface coordination environment [20,21], mitigate defect states and local lattice strain [22,23], and thereby alter the band structure and exciton recombination processes, which ultimately manifests as coupled changes in emission peak position and stability [24,25,26]. However, commonly used long-chain amine ligands [27,28], although beneficial for colloidal stability [29], hinder charge transport and suffer from dynamic desorption [30,31]. Trivalent metal chloride doping has been confirmed to effectively passivate the perovskite surface and enhance its stability [32]. Accordingly, introducing post-treatment ligands with higher reactivity and stronger surface-regulation capability remains of research value.

On this basis, we propose a simple solution-phase post-treatment strategy that broadens the tunable emission range while enhancing stability. With the gradual increase in the amount of TMCS dilute solution added, continuous tuning of the emission wavelength was achieved from 499 nm (TMCS-0) to 480 (TMCS-25), 477 (TMCS-50), 475 (TMCS-75), and 473 nm (TMCS-100). Subsequent analyses by XRD and transmission electron microscopy demonstrated that the morphology and crystal structure of the quantum dots remained unchanged after TMCS treatment. In addition, the appearance of new absorption bands in the FTIR spectra indicated the successful introduction of TMCS and the in situ generation of HCl, which resulted in the etching and passivation of oleic acid ligands on the quantum dot surface. Thermal stability tests further confirmed that TMCS treatment can greatly enhance the thermal stability of the quantum dots.

## 2. Materials and Methods

### 2.1. Methods

#### 2.1.1. Chemicals

Cesium carbonate (Cs_2_CO_3_; Aladdin; 99.9%), lead (II) bromide (PbBr_2_; Aladdin; 99.99%), zinc bromide (ZnBr_2_; Aladdin; 99.99%), trimethylchlorosilane (TMCS; Aladdin; 99%), oleic acid (OA; Aladdin; AR), oleylamine (OAm; Aladdin; 80–90%), 1-octadecene (ODE; Aladdin; >90%), and n-hexane (Aladdin; >99%) were used as received. Unless otherwise specified, reagents labeled “Aladdin” were purchased from Aladdin Reagent (Shanghai) Co., Ltd. (Shanghai, China), and reagents labeled “Macklin” were obtained from Shanghai Macklin Biochemical Co., Ltd. (Shanghai, China).

#### 2.1.2. CsPbBr_3_ Synthesis and Post-Synthetic Ligand Exchange

PbBr_2_ (0.2760 g) and ZnBr_2_ (0.6712 g) were dissolved in a mixed solution containing 30 mL of ODE, 6 mL of OA, and 6 mL of OAm. The resulting solution was degassed under vacuum at 120 °C for 30 min, then switched to a nitrogen atmosphere and heated to 170 °C. A total of 1.6 mL Cs-OA precursor solution was rapidly injected, and the reaction was allowed to proceed for 30 s. The mixture was then quenched by an ice-water bath and cooled to room temperature. The crude nanocrystal dispersion was centrifuged at 10,000 rpm for 10 min, and the supernatant was collected. Excess acetone was added to the supernatant to induce precipitation, followed by centrifugation at 10,000 rpm for 10 min. The precipitate was collected, redispersed in 2 mL of n-hexane, and aliquoted equally into five vials for subsequent ligand exchange. For ligand exchange, a TMCS stock solution was prepared by diluting 0.5 mL of TMCS in 4.5 mL of ODE. Aliquots of 25, 50, 75, and 100 μL of the diluted TMCS solution were added to separate vials of the CsPbBr_3_ QD dispersion and stirred at room temperature for 2 min to complete the ligand exchange process.

### 2.2. Characterization

#### 2.2.1. Transmission Electron Microscopy (TEM)

TEM images were collected using a JEOL JEM-1400 Plus transmission electron microscope (Tokyo, Japan) operated at 120 kV. Samples were prepared by drop-casting a diluted n-hexane suspension onto carbon-coated copper grids.

#### 2.2.2. X-Ray Diffraction (XRD)

XRD patterns were collected on a Bruker D8 ADVANCE diffractometer (Karlsruhe, Germany) using Cu Kα radiation (λ = 1.54056 Å) at 40 kV and 40 mA. Samples were prepared by drop-casting a concentrated n-hexane suspension onto SiO_2_/Si substrates.

#### 2.2.3. Fourier Transform Infrared (FTIR) Spectroscopy

FTIR spectra were recorded at room temperature using a Nicolet 6700 FTIR spectrometer (Thermo Fisher Scientific, Madison, WI, USA). Measurements were collected over 500–4000 cm^−1^. Samples were prepared by drop-casting a highly concentrated n-hexane dispersion of CsPbBr_3_ nanocrystals onto a glass slide, drying under ambient conditions, and then measuring.

#### 2.2.4. Fluorescence Spectrum Measurements

UV–vis absorption spectra were recorded on a Shimadzu UV-1800 spectrophotometer (Kyoto, Japan). Photoluminescence (PL) spectra were measured using a Shimadzu RF-6000 spectrofluorometer (Kyoto, Japan) with excitation wavelengths of 410 nm.

## 3. Results

**Figure 1:**
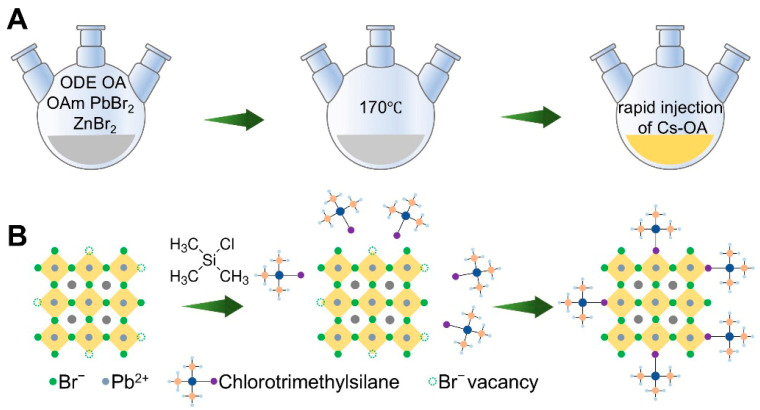
Schematic illustration of CsPbBr_3_ QD synthesis and TMCS ligand exchange. (**A**) Synthesis of CsPbBr_3_ QDs via the hot-injection method. (**B**) Post-treatment ligand exchange.

CsPbBr_3_ QDs were synthesized using a hot-injection method (Figure 1A). During the synthesis, excess ZnBr_2_ was introduced to create a halide-rich environment, thereby regulating the size and improving the synthetic quality of the quantum dots. In the hot-injection process, [PbBr_6_]^4−^ octahedra are first formed in solution. After the Cs precursor is injected, Cs^+^ ions rapidly coordinate with the [PbBr_6_]^4−^ octahedra to generate the CsPbBr_3_ structure. When the halide content in the solution is only three times that of Pb^2+^, the formation of [PbBr_6_]^4−^ octahedra may be insufficient, which can result in halide vacancies. By increasing the halide content in the solution, the formation of [PbBr_6_]^4−^ octahedra is promoted, thus improving the synthesis quality of the CsPbBr_3_ QDs.

To enable controllable post-synthetic tuning of the emission properties, we further implemented a solution-phase TMCS treatment for ligand exchange modification (Figure 1B). Briefly, TMCS was first diluted with ODE. Different volumes of the diluted TMCS solution were then added to the CsPbBr_3_ QD dispersion in n-hexane, followed by stirring at room temperature. The samples were denoted as TMCS-0, TMCS-25, TMCS-50, TMCS-75, and TMCS-100 according to the amount of TMCS solution added. After treatment, the dispersions were stored in sealed vials for subsequent structural and optical characterization as well as thermal-stability measurements.

**Figure 2:**
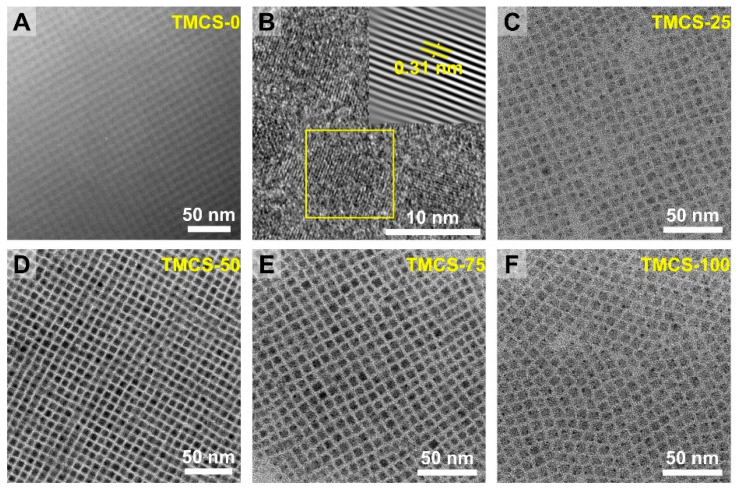
TEM images of the quantum dots for (**A**,**B**) TMCS-0, (**C**) TMCS-25, (**D**) TMCS-50, (**E**) TMCS-75, and (**F**) TMCS-100.

Figure 2 shows the TEM images of the samples before and after treatment. All samples exhibit a cubic structure, and no obvious morphological change is observed after TMCS treatment. Furthermore, we analyzed the size distribution of all samples and found a slight increase in size after TMCS treatment (Figure S1). In particular, the average size of TMCS-75 (Figure S1D) is about 0.4 nm larger than that of the untreated quantum dots, which may be attributed to the passivation of surface defects by TMCS, leading to an increase in the surface thickness.

In the high-resolution image of the TMCS-0 sample, typical features of the cubic phase are observed, with an interplanar spacing of 0.31 nm. In addition, varying degrees of ordered packing can be seen, where QDs in certain local regions form quasi-2D and 3D periodic assemblies. Such periodic packing corresponds to self-assembled superstructures (often referred to as superlattices), which can form when a high-concentration QD dispersion is dried at room temperature as solvent evaporation and interparticle interactions drive organization. Various superlattice configurations of perovskite nanocrystals have been reported, and their formation is closely related to factors such as particle size distribution, the thickness and flexibility of the surface ligand layer, and concentration.

Notably, higher packing regularity is visible in Figure 2A,D,E, appearing brighter and more periodic than in Figure 2C,F. This difference can arise from multiple factors. First, the self-assembly process is inherently stochastic; variations in local concentration and solvent evaporation rate across different regions can lead to fluctuations in ordering. Second, the uniformity of the QD size distribution strongly affects assembly quality, and more monodisperse particles more readily form long-range ordered arrays. Third, an increased stacking thickness along the electron-beam direction enhances projection contrast, making the periodicity appear more ordered. The dark dots in panels C and F are attributed to Pb precipitation induced by prolonged electron-beam irradiation. It should be emphasized that the differences in ordering observed here mainly reflect local self-assembly behavior and are not directly associated with changes in the parent crystal phase. Considering that all samples retain a cubic morphology and good dispersibility, we conclude that the effect of TMCS post-treatment on CsPbBr_3_ QDs primarily occurs at the surface ligand level rather than causing pronounced morphological or structural damage. Because of the small size of the quantum dots, the diffraction peaks are relatively broad (Figure S2A). The peak located at 32.97° is indexed to the (002) plane. After TMCS treatment, partial halide exchange from Br to Cl takes place. Owing to the smaller ionic radius of Cl^−^, the interplanar spacing is reduced, causing the diffraction peak to shift to a higher diffraction angle (Figure S2B).

**Figure 3:**
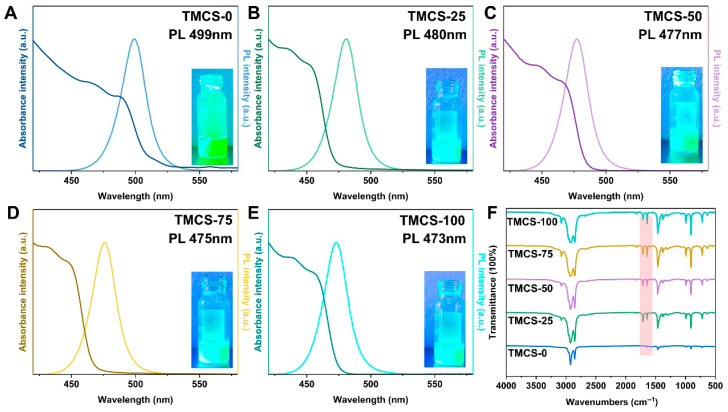
Photoluminescence (PL), UV–vis absorption spectra and FTIR spectra for (**A**) TMCS-0, (**B**) TMCS-25, (**C**) TMCS-50, (**D**) TMCS-75, and (**E**) TMCS-100. (**F**) FTIR spectra.

All samples exhibit narrow PL and UV–vis absorption features, indicative of small emission linewidths and thus high color purity (Figure 3A–E). With increasing ligand dosage, the emission peak shows a clear ligand-molarity dependence: the PL maximum continuously shifts from 499 nm to 473 nm as the TMCS amount increases, demonstrating a systematic and monotonic wavelength modulation. The inset photograph shows the corresponding quantum dot solutions under UV illumination, in good agreement with the PL emission behavior. The UV–vis absorption peak of the TMCS-0 sample is centered at 489 nm, with a Stokes shift of 10 nm. Following TMCS treatment, a blue shift of the absorption peak is observed, together with a further increase in the Stokes shift. These changes may be related to halide exchange or surface dielectric modification; however, the detailed mechanism is still not fully understood.

To further verify the surface-chemical changes induced by TMCS post-treatment, FTIR spectroscopy was performed on samples prepared with different TMCS dosages (Figure 3F). Compared with the pristine sample (TMCS-0), the treated samples display new absorption bands primarily located at 1820 cm^−1^, 1260 cm^−1^ and 850 cm^−1^, and their intensities progressively increase with increasing TMCS addition. The absorption peak at 1820 cm^−1^ lies in the characteristic region of acyl chlorides and is assigned to RC(=O)Cl (1800–1850 cm^−1^), indicating that TMCS reacts with oleic acid to generate HCl in situ (Figure 3F). Owing to the low TMCS content in the samples, the characteristic Si-CH_3_-related absorption bands are much weaker than those of oleic acid and oleylamine and can only be clearly identified in the enlarged spectrum (Figure S3). The peaks at 1260 cm^−1^ and 850 cm^−1^ (Figure S3) are attributed to the symmetric deformation vibration of Si-CH_3_ and the Si-C or Si-CH_3_ rocking/stretching-related vibrations (850–750 cm^−1^), respectively, further confirming the successful introduction of TMCS. Moreover, the intensities of these peaks gradually increase with increasing TMCS content.

Meanwhile, characteristic bands at 2853 cm^−1^, 1641 cm^−1^, 1467 cm^−1^, 992 cm^−1^, 909 cm^−1^, and 721 cm^−1^ become stronger overall. Specifically, the bands at 2853 cm^−1^ and 1467 cm^−1^ correspond to the stretching and bending vibrations of long-chain alkyl CH_2_ groups, respectively, and the band at 721 cm^−1^ is assigned to the rocking vibration of long-chain (CH_2_) segments. The bands at 1641 cm^−1^, 992 cm^−1^, and 909 cm^−1^ are associated with vibrations of unsaturated bonds (C=C) and =C-H modes. Collectively, these spectral changes indicate that TMCS post-treatment markedly alters the organic coordination environment at the quantum dot surface, consistent with ligand exchange, re-coordination, and reconstruction of the surface organic layer. Notably, at higher TMCS dosages, stronger byproduct-related signatures may emerge, which could be detrimental to surface passivation.

**Figure 4:**
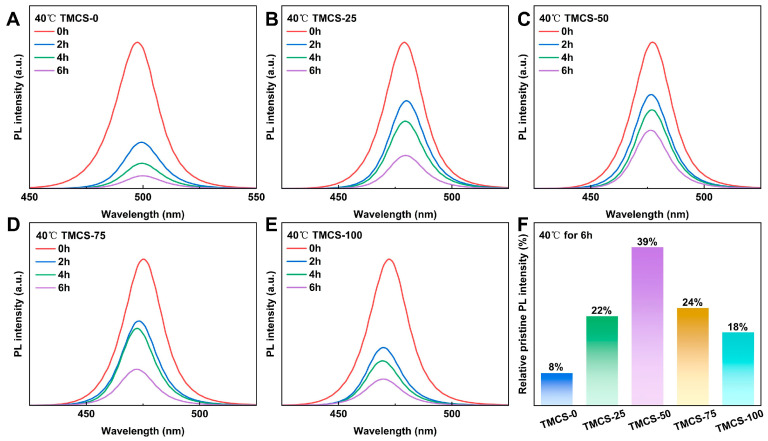
Thermal stability test at 40 °C for 2, 4, and 6 h for (**A**) TMCS-0, (**B**) TMCS-25, (**C**) TMCS-50, (**D**) TMCS-75, and (**E**) TMCS-100. (**F**) Comparison of the PL intensities of all samples after heating at 40 °C for 6 h relative to their initial values.

To evaluate the influence of TMCS post-treatment on the thermal stability of the samples, the QDs were dispersed in n-hexane and maintained on a heating plate at 40 °C for thermal-stability testing (Figure 4). With prolonged heating time, the PL intensities of all samples showed a pronounced decline. After heating for 6 h, the PL intensity of the untreated sample remained at only 8% of its initial value, whereas all TMCS-treated samples exhibited markedly improved PL intensity retention (Figure 4F). Notably, TMCS-50 retained 39% of its initial PL intensity after 6 h of heating, indicating the best thermal stability among all samples. Additionally, the quantum dot solutions were spin-coated onto silicon substrates, and photographs of their luminescence under 365 nm UV irradiation at different temperatures were recorded (Figure S4). The untreated sample showed no visible emission at 140 °C, whereas the TMCS-treated samples retained bright luminescence at higher temperatures. In particular, the TMCS-25, TMCS-50, and TMCS-75 samples still exhibited weak emission at 180 °C. Notably, the thermal stability of TMCS-100 was lower than that of TMCS-50, in good agreement with the quantitative results in Figure 4. This behavior may be associated with an excessive amount of TMCS. FTIR results suggest that TMCS can react with oleic acid to generate HCl in situ, and excess HCl may further deteriorate the quantum dots. However, the exact mechanism remains unclear and requires further detailed characterization. Overall, an appropriate TMCS treatment significantly enhances the thermal stability of the QDs.

## 4. Discussion

In this work, a TMCS solution post-treatment strategy was developed to modify the surface of CsPbBr_3_ QDs using reactive ligands, thereby achieving the synergistic enhancement of their photoluminescent properties and thermal stability. TMCS can react with oleic acid to generate HCl in situ, which subsequently etches and passivates the quantum dot surface. This observation further suggests that TMBS and TMIS may also induce similar surface etching and passivation effects. When the halide species introduced by the ligand is the same as that originally present in the quantum dots, such post-treatment ligand exchange is expected to passivate surface defects and improve the stability of the quantum dots. By contrast, when the halide species in the added ligand differs from that of the quantum dots, partial halide exchange can be achieved. In addition, the newly introduced halide is closely associated with the ligand, which may further enhance the phase stability of mixed-halide quantum dots and suppress phase segregation under operational conditions.

## Data Availability

The original contributions presented in this study are included in the article/Supplementary Materials. Further inquiries can be directed to the corresponding author.

## Supplementary Materials

The following supporting information can be downloaded at: https://www.mdpi.com/article/10.3390/ma19091860/s1, Figure S1: Size distribution statistics for (A) TMCS-0, (B) TMCS-25, (C) TMCS-50, (D) TMCS-75, and (E) TMCS-100; Figure S2: XRD patterns of the quantum dots; Figure S3: Enlarged view of the FTIR spectra; Figure S4: Photographs of the QDs emitting under UV illumination at different temperatures.
