# Supplementary material for: Tunable Emission Peak Position and Enhanced Thermal Stability of CsPbBr3 Quantum Dots via TMCS Ligand Exchange
Source: Materials (Basel). 2026 May 1;19(9):1860. doi: 10.3390/ma19091860 (PMC13165425; doi:10.3390/ma19091860)
Supplement: Supplementary file 1 [file materials-19-01860-s001.zip › materials-4242114-supplementary.pdf]

Tunable Emission Peak Position and Enhanced Thermal Stability of $CsPbBr_3$ Quantum Dots via TMCS Ligand Exchange

Chong Peng [†], Yutao Feng [†], Zhicheng Shen [†], Zhe Pang, Yingfei Liu, JiaQian Que, Kefeiyang Hu, Xingbo Huang and Yong Liu [*]

State Key Laboratory of Advanced Technology for Materials Synthesis and Processing, International School of Materials Science and Engineering (ISMSE), Wuhan University of Technology, Wuhan 430070, China

[†]These authors contributed equally to this work.

*Correspondence: liuyong3873@whut.edu.cn

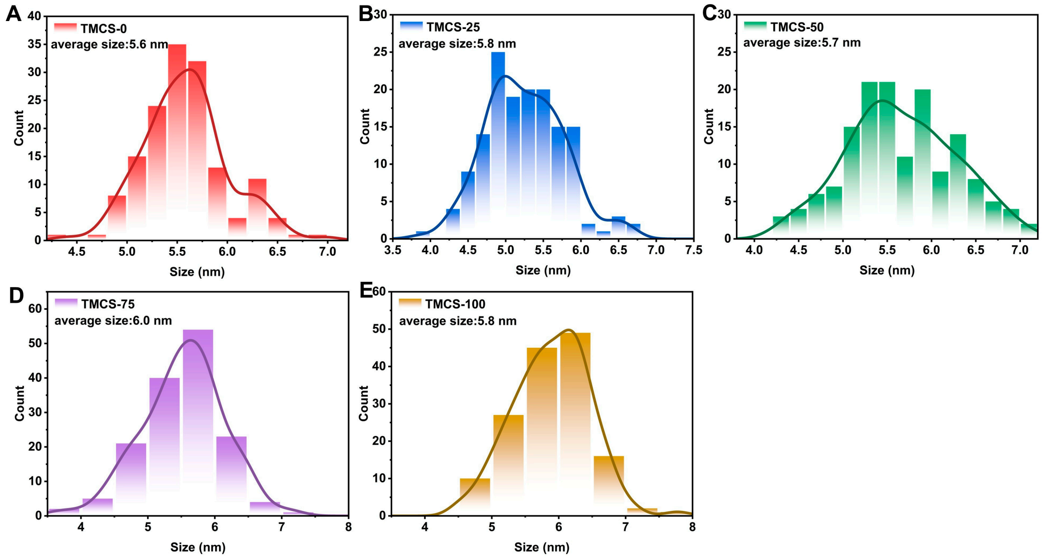

Figure S1. Size distribution statistics for (A) TMCS-0, (B) TMCS-25, (C) TMCS-50, (D) TMCS-75, and (E) TMCS-100.

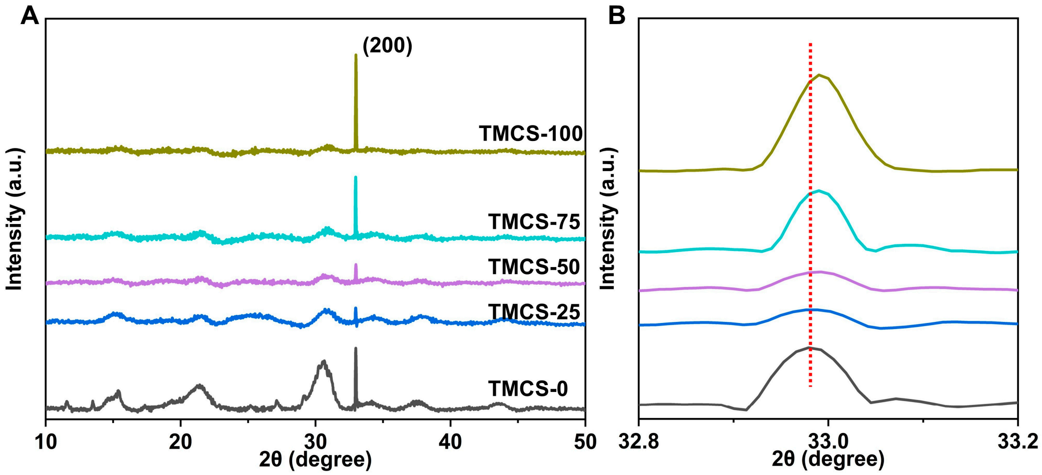

Figure S2. XRD patterns of the quantum dots.

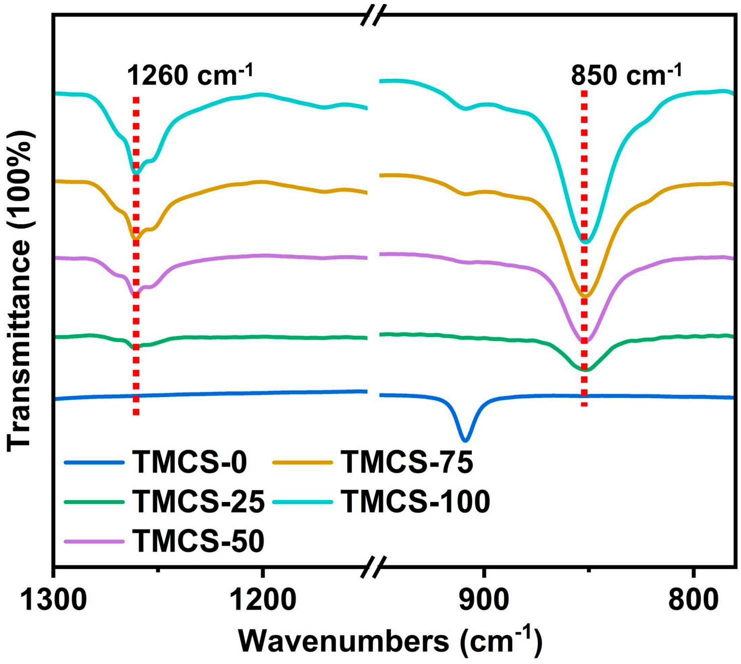

Figure S3. Enlarged view of the FTIR spectra.

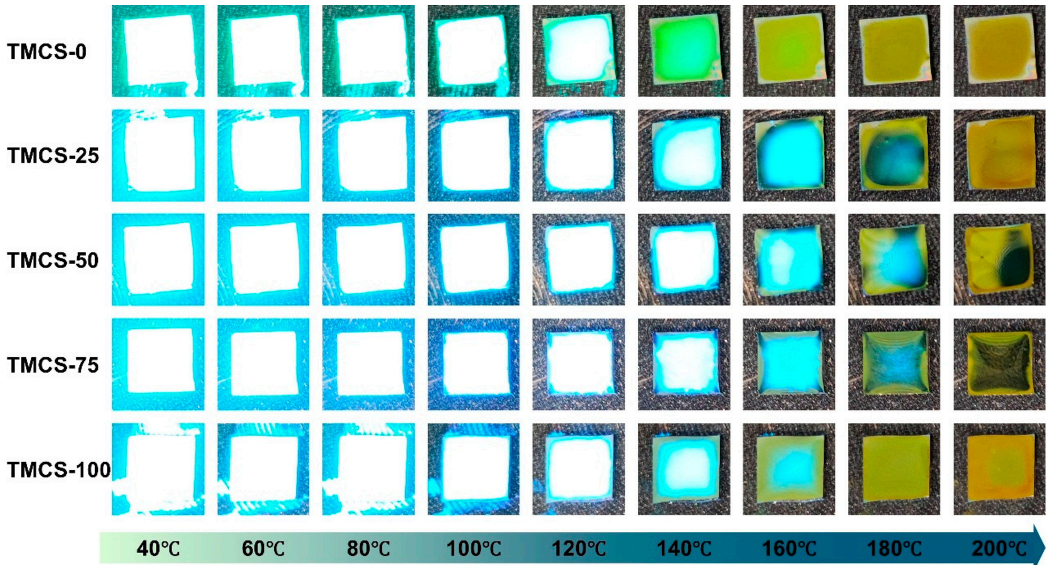

Figure S4. Photographs of the QDs emitting under UV illumination at different temperatures.
